# Visual outcomes and patient satisfaction 1 and 12 months after combined implantation of extended depth of focus and trifocal intraocular lenses

**DOI:** 10.1007/s10792-021-01970-3

**Published:** 2021-09-21

**Authors:** Richard N. McNeely, Salissou Moutari, Stephen Stewart, Jonathan E. Moore

**Affiliations:** 1 Cathedral Eye Clinic, 89-91 Academy Street, Belfast, BT1 2 LS Northern Ireland UK; 2 School of Mathematics and Physics, Queens University Belfast, Belfast, Northern Ireland UK; 3 Biomedical Sciences Research Institute, University of Ulster, Coleraine, Northern Ireland UK; 4 Tianjin Medical University, Tianjin, China; 5 Aston University, Birmingham, UK

**Keywords:** Quality of vision, Cataract surgery, Refractive lens exchange, Visual outcomes, Extended depth of focus IOL, Trifocal IOL

## Abstract

**Purpose:**

To assess the 1-month and 12-month postoperative visual performance and subjective outcomes following combined implantation of an extended depth of focus (EDOF) intraocular lens (IOL) and a trifocal IOL.

**Methods:**

The study enrolled consecutive patients undergoing refractive lens extraction or cataract surgery with combined implantation of an EDOF IOL (dominant eye) and trifocal IOL. Uncorrected (UDVA) and best-corrected (CDVA) distance visual acuities, uncorrected intermediate (UIVA) and near (UNVA) visual acuities, and subjective questionnaires were evaluated 1 month and 12 months postoperatively.

**Results:**

The study enrolled 58 consecutive patients. Binocular UDVA, UIVA and UNVA were − 0.08 ± 0.07 logMAR, 0.15 ± 0.14 logMAR and 0.17 ± 0.11 logMAR at 1 month, compared to − 0.09 ± 0.06 logMAR (*P* = .323), 0.11 ± 0.10 logMAR (*P* = .030) and 0.13 ± 0.10 logMAR (*P* = 0.008) at 12 months. Satisfaction was high with 93.1% of patients fulfilled or more than fulfilled postoperatively, and 84.5% and 86.3% reported spectacle independence for near at the respective postoperative assessments. The mean daytime and nighttime quality of vision (QoV) scores were 9.12 ± 0.94 and 7.88 ± 1.74 at 1 month, compared to 9.24 ± 0.78 (*P* = .183) and 8.26 ± 1.38 (*P* = .043) at 12 months.

**Conclusions:**

This IOL combination provides good unaided visual acuity at 1 and 12 months postoperatively, with high functional vision and postoperative satisfaction reported at 1 and 12 months. However, a significant improvement in overall nighttime QoV at the 12 months assessment was found.

## Introduction

Various methodologies have been introduced in modern day lens-based surgery to provide a range of clear vision [1–6] with the least amount of visual side effects. Our recent study [7] sought to outline the clinical and patient-reported outcomes at an early postoperative period of the latest extended depth of focus (EDOF) technology, thought to provide superior intermediate vision with fewer unwanted visual phenomena, implanted in combination with a trifocal intraocular lens (IOL). The EDOF IOL was used in the dominant eye to provide good distance, intermediate and improve near vision and produce fewer unwanted visual phenomena, with the trifocal IOL implanted in the nondominant eye to provide adequate reading vision. This recent study [7] reported that the combination of an EDOF and trifocal IOL provides good unaided visual acuity for distance, and near distances, providing high postoperative satisfaction and functional vision, at an early postoperative stage. This current study sought to outline the objective visual and refractive outcomes, and patient satisfaction of this IOL combination in a cohort of patients at a longer postoperative period of 1 year, and to determine whether the objective and subjective outcomes altered over this postoperative period.

## Methods

This retrospective study recruited patients who received a combined implantation of an EDOF IOL and a trifocal IOL following refractive lens exchange or cataract surgery between March 2018 and March 2019.

Consecutive patients were enrolled in this study with each patient giving their informed consent to undergo refractive lens exchange or cataract surgery prior to surgery, and for their unidentifiable patient data to be used for audit and publication.

The exclusion criteria for this study were any neuro-ophthalmic disease, corneal surgery or disease, ocular inflammation, a history of retinal detachment or glaucoma and macular disease. Preoperative corneal astigmatism of 1.50 diopters (*D*) or less, no previous refractive surgery and the absence of any other ocular pathology were required with each patient for inclusion in this current study. Patients who developed visually significant posterior capsular opacification (PCO) and who had not yet received neodymium:yttrium–aluminum-garnet (Nd:YAG) at their 1 year assessment were also excluded.

All patients received full ophthalmological assessment preoperatively. Uncorrected (UDVA) and corrected (CDVA) distance visual acuities were evaluated with logarithmic acuity (logMAR) charts, and uncorrected intermediate (UIVA) and near (UNVA) visual acuities were evaluated with Radner reading charts (70 cm and 40 cm). Slit-lamp examination, Goldmann tonometry, and dilated funduscopy were completed. Additionally, stereopsis (TNO stereo test), corneal topography (OPD-Scan II; NIDEK Co., Ltd., Gamagori, Japan), corneal tomography (Pentacam, Oculus Optikgeäte GmbH) and retinal optical coherence tomography (Cirrus 4000 OCT; Carl Zeiss Meditec) were completed. Biometry was completed with the Aladdin (Topcon). For each case the SRK/T, Haigis, HofferQ and Barrett Universal II were compared, and a decision made regarding the IOL power. To determine ocular dominance, the pointing methodology was utilized. Patients were asked to visually align their finger with a spot light source at 6 m with each eye then occluded. Ocular dominance was determined as the eye showing the smallest separation between the finger and the light source. Patients were also asked which eye they would use for sighting a camera and a rifle. The results from both ocular dominance assessments were required to be consistent to confirm ocular dominance. In the presence of significantly reduced vision due to cataract, the eye with the most severe cataract was operated on first.

Patients were examined 1 month and 12 months postoperatively. These assessments included manifest refraction, UDVA, CDVA, UIVA and UNVA. Patients also completed a purpose developed quality of vision (QoV) questionnaire as previously outlined [7]. This questionnaire utilizes pictures to aid understanding of the questions and patients report their responses on a Likert scale. To gain a better overall understanding of each patients’ overall satisfaction, patients were asked to rate their day and night QoV on a linear 0 to 10 scale. A purpose-developed satisfaction questionnaire was also utilized where patients report their satisfaction regarding their distance, intermediate and near vision, and their overall vision, as outlined previously [7].

### Intraocular lens

The AT LARA 829MP IOL (AT LARA 829MP; Carl Zeiss Meditec, Jena, Germany) is an EDOF IOL made from hydrophilic acrylic with hydrophobic surface properties and has a 6 mm optic size and a 11 mm overall length. The IOL is an aspheric lens based on a diffractive principle with a chromatic aberration-correcting and aberration-neutral optical design. There are two additions of + 0.95 D and + 1.90 D with a light bridge optical design and Smooth Microphase (SMP) technology to minimize light scattering. The available powers are − 10.00 to + 32.00 D in 0.50 D increments.

The AT LISA tri 839MP IOL (AT LISA tri839MP; Carl Zeiss Meditec, Jena, Germany) is a diffractive trifocal IOL with + 1.66 D and + 3.00 D addition powers for intermediate and near vision. The IOL is made from hydrophilic acrylic with hydrophobic surface properties and has a 6 mm optic size and a 11 mm overall length. It is available in powers between 0.00 and + 32.00 D in 0.50 D increments.

### Surgical technique

All surgeries were performed, under Sub-Tenon or topical anesthesia, by the same experienced surgeon (J.E.M). The first eye received an EDOF IOL in the dominant eye followed by the trifocal in the fellow eye one week later and emmetropia was the refractive aim in each case. Following phacoemulsification the IOL was inserted into the capsular bag through an incision of 2.75 mm. A capsular tension ring was implanted in each case.

### Statistical analysis

Statistical analysis was performed using SPSS for Windows (Statistical Package for the Social Sciences, Version 25, Chicago, Illinois, USA) and Excel (Microsoft; Redmond, Washington, USA). The preoperative and postoperative parameters were presented as means and standard deviations or percentages. The Kolmogorov–Smirnov test was used to assess normality. When assessing continuous normal data, the Student’s paired *t* test was used to compare postoperative data, and when assessing nonparametric data, the Wilcoxon rank-sum test was used. For all statistical analysis, the level of significance was *P* < 0.05.

## Results

This study included 116 eyes of 58 patients with a mean age of 59.0 ± 7.0 (44–81) years. Table 1 outlines the demographics and the preoperative clinical data.

**Table 1:** Demographics and clinical data

Parameter	Preoperative	
	AT LARA	AT LISA
No. of patients (eyes)	58 (58)	58 (58)
Age (y), mean ± SD (range)	59 ± 7.0 (44−81)	
Gender, male / female (%)	19/39 (32.8/67.2)	
Myopia / hyperopia (%)	9/49 (15.5/84.5)	
Axial length (mm), mean ± SD (range)	23.54 ± 1.11 (21.47−27.27)	23.59 ± 1.17 (20.98−27.35)
Power of implanted IOL (D), mean ± SD (range)	20.76 ± 3.43 (9.5 to 27.5)	21.51 ± 3.97 (7.0 to 29.0)
Clinical, mean ± SD (range)		
Sphere (D)	0.88 ± 2.77 (− 10−4.50)	1.00 ± 2.99 (− 12−5.25)
Cylinder (D)	− 0.48 ± 0.45 (− 2−0)	− 0.54 ± 0.52 (− 2.25−0)
MSE (D)	0.63 ± 2.85 (− 10.63−3.88)	0.72 ± 3.06 (− 12.63−4.50)
CDVA	− 0.01 ± 0.28 (− 0.2−1.85)	− 0.02 ± 0.20 (− 0.2−1.20)

SD = standard deviation; IOL = intraocular lens; D = diopters; MSE = manifest spherical equivalent; CDVA = corrected distance visual acuity

### Visual acuity and refraction

**Fig. 1:**
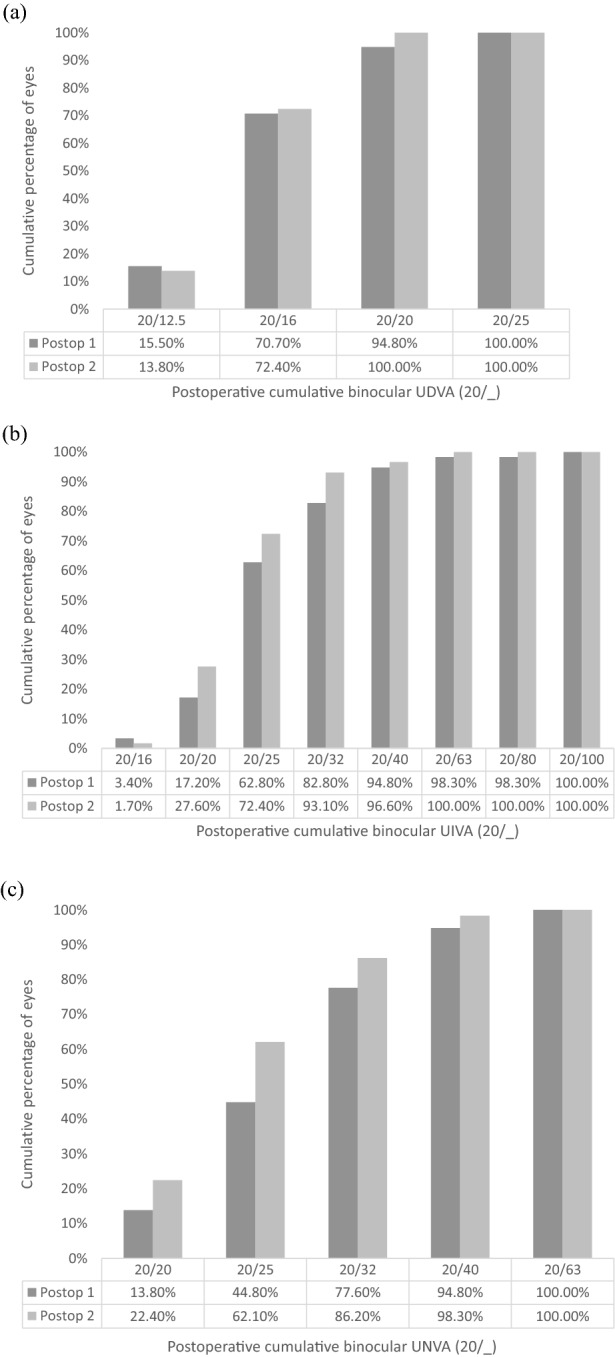
Cumulative binocular uncorrected **a** distance, **b** intermediate, and **c** near visual acuity 1 month and 12 months postoperatively. (UDVA = uncorrected distance visual acuity; CDVA = uncorrected distance visual acuity)

**Fig. 2:**
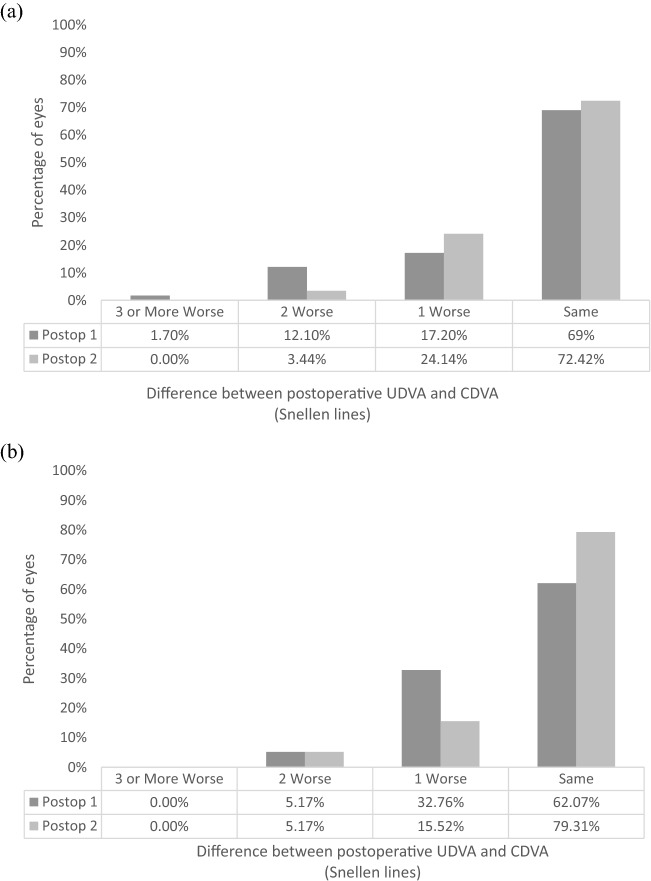
Histogram showing the efficacy of lines of difference between postoperative UDVA and CDVA for **a** EDOF IOL and **b** trifocal IOL, at 1 month and 12 months postoperatively. (UDVA = uncorrected distance visual acuity, CDVA = corrected distance visual acuity)

**Fig. 3:**
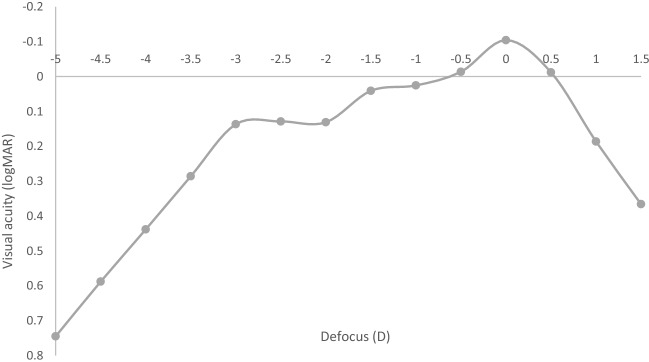
Binocular defocus curve at 1 month. D = diopters

Figure 1 outlines the postoperative binocular UDVA, UIVA and UNVA at 1 month and 12 months postoperatively. Table 2 outlines a comparison of the objective visual and refractive outcomes between the two postoperative assessments. A significant difference between the two postoperative assessments was found in binocular UIVA (*P* = 0.030) and UNVA (*P* = 0.008), with an improvement found at 12 months at both distances. Figure 2 shows the difference between the postoperative UDVA and CDVA at 1 and 12 months postoperatively for the EDOF and trifocal IOL eyes. Figure 3 displays the mean binocular defocus curve found at the first postoperative assessment.

**Table 2:** Comparison of 1-month and 12-month postoperative clinical data

Parameter, mean ± SD (range)	Postoperative 1		Postoperative 2		*P* Value	
	AT LARA	AT LISA	AT LARA	AT LISA	AT LARA	AT LISA
	58 (58)	58 (58)	58 (58)	58 (58)	58 (58)	58 (58)
Sphere (D)	0.48 ± 0.43 (− 0.50−1.75)	0.31 ± 0.40 (− 0.50 −1.50)	0.50 ± 0.40 (0 −1.75)	0.34 ± 0.41 (− 0.50 −1.50)	0.766	0.592
Cylinder (D)	− 0.25 ± 0.34 (− 1.50 −0)	− 0.32 ± 0.37 (− 1.50 −0)	− 0.25 ± 0.31 (− 1.50 −0)	− 0.30 ± 0.36 (− 1.25 −0)	0.805	0.709
MSE (D)	0.36 ± 0.37 (− 0.50 −1.50)	0.16 ± 0.34 (− 0.88 −0.88)	0.37 ± 0.35 (− 0.25 −1.13)	0.19 ± 0.37 (− 0.50 −1.00)	0.796	0.416
UDVA (logMAR)	− 0.01 ± 0.11 (− 0.20 −0.24)	0 ± 0.09 (− 0.20 −0.24)	− 0.02 ± 0.09 (− 0.20 −0.20)	− 0.02 ± 0.09 (− 0.20 −0.20)	0.439	0.315
Binocular UDVA (logMAR)	− 0.08 ± 0.07 (− 0.20 −0.10)		− 0.09 ± 0.06 (− 0.20 −0)		0.323	
UIVA (logMAR)	0.18 ± 0.16 (− 0.10 −0.70)	0.23 ± 0.14 (− 0.10 −0.70)	0.17 ± 0.12 (0 −0.60)	0.19 ± 0.12 (0 −0.60)	0.513	0.128
Binocular UIVA (logMAR)	0.15 ± 0.14 (− 0.10 −0.70)		0.11 ± 0.10 (− 0.10 −0.40)		0.030	
UNVA (logMAR)	0.37 ± 0.16 (0.10 −0.80)	0.19 ± 0.12 (0 −0.50)	0.38 ± 0.15 (0.10 −0.70)	0.18 ± 0.14 (0 −0.50)	0.802	0.927
Binocular UNVA (logMAR)	0.17 ± 0.11 (0 −0.50)		0.13 ± 0.10 (0 −0.40)		0.008	
CDVA	− 0.08 ± 0.06 (− 0.20 −0.10)	− 0.06 ± 0.08 (− 0.20 −0.10)	− 0.07 ± 0.07 (− 0.20 −0.10)	− 0.07 ± 0.08 (− 0.20 −0.12)	0.619	0.699

SD = standard deviation; D = diopters; MSE = manifest spherical equivalent; UDVA = uncorrected distance visual acuity; UIVA = uncorrected intermediate visual acuity; UNVA = uncorrected near visual acuity; CDVA = corrected distance visual acuity

**Fig. 4:**
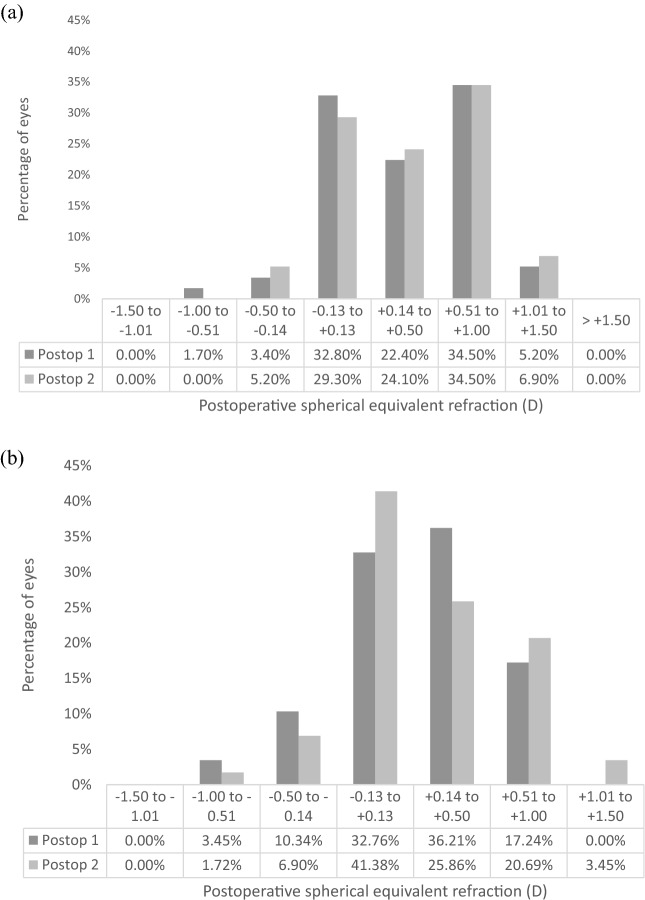
Predictability for **a** EDOF IOL and **b** trifocal IOL, at 1 month and 12 months postoperatively. (SE = spherical equivalent; D = dioptres)

**Fig. 5:**
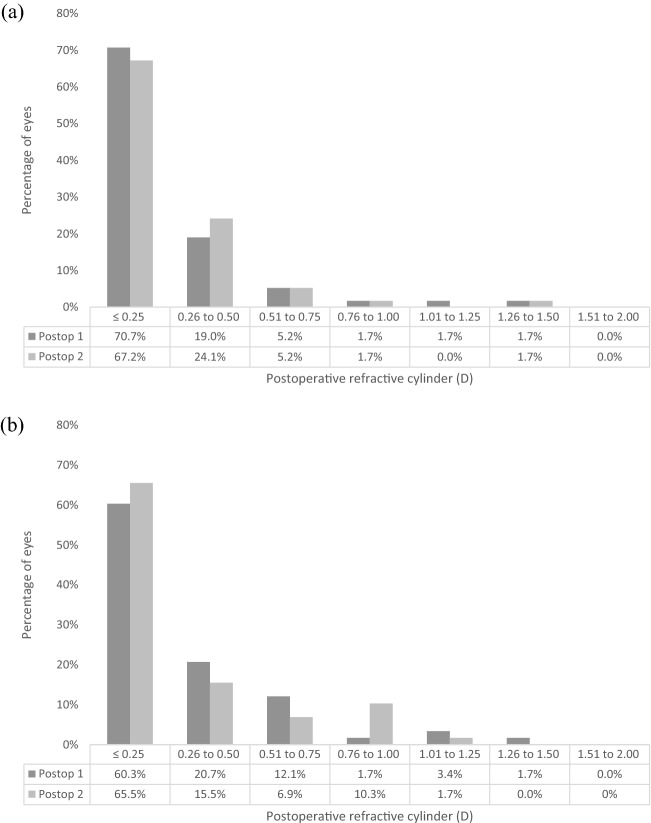
Postoperative refractive cylinder for **a** EDOF IOL and **b** trifocal IOL, at 1 month and 12 months postoperatively

**Fig. 6:**
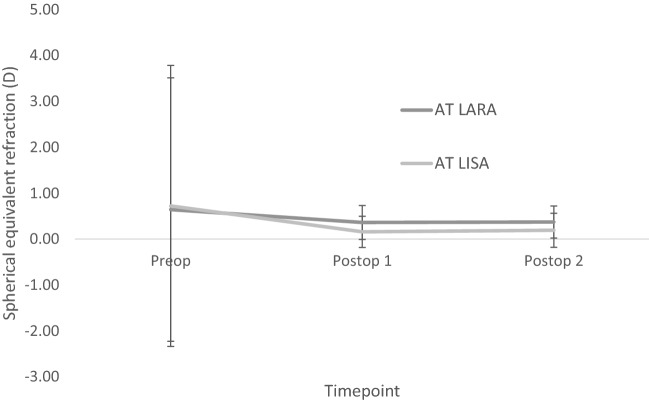
Stability up to 12 months postoperatively plotted as the mean ± SD of the SE refraction, for both IOL designs

Figure 4a shows the predictability for the EDOF IOLs. Postoperatively, 58.6% of EDOF IOLs eyes were within ± 0.50 D of the refractive target at 1 month and 12 months. One month postoperatively, 79.3% of trifocal IOLs were within ± 0.50 D, and 74.1% at 12 months (Fig. 4b). Figure 5 outlines the postoperative refractive cylinder at both postoperative assessments for the respective IOL designs, where 89.7% and 91.3% of EDOF IOL eyes had refractive cylinder of 0.5 D or less at the two respective postoperative assessments, and 81% of trifocal IOL eyes at both postoperative assessments. There was no significant difference in mean refractive cylinder with both IOL designs (Table 2). Figure 6 shows the refractive stability of the spherical equivalent refraction up to 12 months postoperatively. Fifty-seven (98.3%) EDOF IOL eyes and all trifocal IOL eyes had a change of spherical equivalent refraction of 1.00 D or less between 1 and 12 months postoperatively. Table 3 outlines how the postoperative refractive error changes between 1 and 12 months. With the EDOF and trifocal IOLs 75.7% and 58.9% of eyes changed less than ± 0.25 D between the two postoperative assessments. For the EDOF IOL 13.8% of eyes displayed a myopic shift of > 0.25 D compared to 10.3% eyes having a hyperopic shift. With the trifocal IOL 16.1% of eyes had a myopic shift of > 0.25 D compared to 25% showing a hyperopic shift.

**Table 3:** Change in refractive error between 1 and 12 months postoperatively for the EDOF IOL and trifocal IOLs

	No change SE (within ± 0.25)	% myopic 0.26 −0.50	% myopic 0.51 −1.00	% myopic > 1.00	% hyperopic 0.26 −0.50	% hyperopic 0.51 −1.00	% hyperopic > 1.00
EDOF IOL (*n* = 58 eyes)	75.9 (*n* = 44)	6.9 (*n* = 4)	6.9 (*n* = 4)	−	3.4 (*n* = 2)	5.2 (*n* = 3)	1.7 (n = 1)
Trifocal IOL (*n* = 56 eyes)	58.9 (*n* = 33)	14.3 (*n* = 8)	1.8 (*n* = 1)	−	19.6 (*n* = 11)	5.4 (*n* = 3)	−

### Patient-reported outcomes

Table 4 outlines the responses from a patient satisfaction questionnaire, and Table 5 shows the subjective responses to visual disturbances and photopic phenomena at 1 and 12 months.

**Table 4:** Comparison of 1-month and 12-month patient experience postoperative data

Postoperative assessment	Question				
	Compare your vision preoperative to postoperative				
	Better	Unchanged	Worse	Can’t remember	
Postop 1	94.8%	3.5%	1.7%	0%	
Postop 2	100%	0%	0%	0%	
	Would you choose this procedure again?				
	Yes	No	Maybe		
Postop 1	94.9%	1.7%	3.4%		
Postop 2	98.3%	0%	1.7%		
	Would you recommend this procedure?				
	Yes	No	Maybe		
Postop 1	94.9%	1.7%	3.4%		
Postop 2	98.3%	0%	1.7%		
	How often do you require reading glasses?				
	Never	Occasionally	Quite often	Always	
Postop 1	84.5%	13.8%	1.7%	0%	
Postop 2	86.3%	10.3%	3.4%	0%	
	How much difficulty do you have doing a regular task that requires you to see well in the distance?				
	Distance vision is clear	Slight problem	Moderate problem	Severe problem	Intolerable problem
Postop 1	89.7%	6.9%	0%	3.4%	0%
Postop 2	89.7%	10.3%	0%	0%	0%
	How much difficulty do you have doing a regular task that requires you to see well at intermediate working distances?				
	Intermediate vision is clear	Slight problem	Moderate problem	Severe problem	Intolerable problem
Postop 1	86.2%	8.6%	5.2%	0%	0%
Postop 2	89.6%	5.2%	5.2%	0%	0%
	How much difficulty do you have doing a regular task that requires you to see well at near working distances?				
	Near vision is clear	Slight problem	Moderate problem	Severe problem	Intolerable problem
Postop 1	75.9%	19.0%	3.4%	1.7%	0%
Postop 2	77.6%	19.0%	3.4%	0%	0%
	How were your expectations fulfilled with the procedure?				
	More than fulfilled	Fulfilled	Sufficiently fulfilled	Not fulfilled at all	
Postop 1	43.1%	50.0%	5.2%	1.7%	
Postop 2	44.8%	48.3%	6.9%	0%

**Table 5:** Comparison of 1-month and 12-month postoperative visual phenomena data

	Postop 1	Postop 2	P Value
Glare	0.53 ± 0.92 (0, 3)	0.43 ± 0.75 (0, 3)	.498
Haloes	0.64 ± 0.87 (0, 3)	0.47 ± 0.68 (0, 2)	.176
Starburst	0.31 ± 0.75 (0, 3)	0.29 ± 0.62 (0, 2)	.903
Hazy vision	0.14 ± 0.44 (0, 2)	0.03 ± 0.18 (0, 1)	.063
Blurred vision	0.16 ± 0.45 (0, 2)	0.14 ± 0.44 (0, 2)	.709
Distortion	0.02 ± 0.13 (0, 1)	0	.317
Double vision	0.05 ± 0.29 (0, 2)	0.03 ± 0.26 (0, 2)	.317
Vision Fluctuate	0.17 ± 0.42 (0, 2)	0.21 ± 0.45 (0, 2)	.317
Depth perception	0.10 ± 0.36 (0, 2)	0.10 ± 0.36 (0, 2)	1.000
QoV Day	9.12 ± 0.94 (7, 10)	9.24 ± 0.78 (7, 10)	.183
QoV Night	7.88 ± 1.74 (2, 10)	8.26 ± 1.38 (5, 10)	.043

Visual phenomena calculated on a scale 0 (not at all) to 3 (very). Values represent mean ± SD (range); QoV is calculated on a scale 0 (worst) to 10 (best). Values represent mean ± SD (range)

A significant improvement in overall nighttime QoV was found at 12 months when compared to the early postoperative assessment (*P* = 0.043).

### Complications

Eight eyes (6.7%) required neodymium:YAG (Nd:YAG) capsulotomy. Furthermore, two eyes implanted with a trifocal IOL required further laser enhancement with laser in situ keratomileusis (LASIK) for residual refractive error. No other adverse events occurred.

## Discussion

Multifocal IOLs are well recognized as a method to provide high patient satisfaction and spectacle independence following cataract surgery or refractive lens exchange; however visual phenomena can be present postoperatively which can cause dissatisfaction [8, 9], and in some cases an exchange of the IOL is required [10]. New designs of multifocal IOLs are continuing to be developed to optimize postoperative outcomes and reduced unwanted side effects. Our recent study outlined the early postoperative outcomes found after implantation of a new EDOF IOL in combination with a trifocal IOL [7]. This present study sought to determine the objective and subjective outcomes after this bilateral implantation of an EDOF IOL and a trifocal IOL up to a longer postoperative timepoint, and determine how these outcomes alter, if at all, over this postoperative period.

This IOL combination displayed excellent UDVA at both postoperative periods. The mean binocular UDVA was − 0.08 ± 0.07 logMAR at 1 month and − 0.09 ± 0.06 logMAR at 12 months, and no significant difference was found between the two postoperative periods (Table 2). This is superior to that found in the early postoperative outcomes following bilateral implantation of the new EDOF IOL [11], and that found in another study of the same implantation methodology 3 months postoperatively [12]. Furthermore, UDVA appears to be superior to that found in bilateral trifocal IOL implantation [13]. To our knowledge there is no study outlining the outcomes of this combination up to 1 year and this current study shows that the binocular UDVA remains stable throughout the first postoperative year.

The binocular UIVA results found were 0.15 ± 0.14 logMAR and 0.11 ± 0.10 logMAR at the two respective assessments (Table 2). The 1 month binocular UIVA is similar to that found in a study following bilateral implantation of a rotationally asymmetric multifocal IOL [14]. Similarly, this implantation methodology showed excellent binocular UNVA with a mean value of 0.17 ± 0.11 logMAR at 1 month and 0.13 ± 0.10 logMAR at 12 months, which appears to be superior to that found in bilateral implantation of trifocal IOLs [15], and bilateral EDOF IOLs [11, 16], however similar to that found in a study of rotationally asymmetric multifocal IOLs [17]. Both the binocular UIVA and UNVA showed significantly better outcomes at 12 months when compared to the early postoperative assessment. However, the difference does not appear to be clinically significant with only 0.04 logMAR improvement. Furthermore, when patients were asked directly about the quality of their intermediate and near vision, and their ability to perform daily tasks at these distances, there was no significant difference in both the intermediate (*P* = 0.773) and near (*P* = 0.527) vision responses between the two postoperative assessments (Table 4). This study highlights that a range of clear unaided visual acuity is achieved by this implantation methodology and is maintained up to 12 months postoperatively. Furthermore, Fig. 3 displays the defocus curve for this IOL combination, where a peak visual acuity was found in the distance then a gradual decrease to a viewing distance of 50 cm. Visual acuity then appears to remain stable from 50 to 33 cm.

As found in our previous study [7], there is a hyperopic tendency at both postoperative assessments with the EDOF IOL (Fig. 4), which may be due keratometric changes or to the IOL moving posteriorly due to capsular contractions [18, 19], and direct assessment of IOL shift would further help explain the cause. Adjustments have since been made to the biometry A-constants to optimize the postoperative refractive outcomes in our clinic. A study showed that with optical biometry, an improvement of eyes within ± 1.00D of the refractive target was noted when using optimized A-constants [20, 21]. Therefore, optimized A-constants are now utilized in our clinic. However, the difference in refractive outcomes is not significantly different between the postoperative assessments (Table 2) with a good refractive stability outlined in Fig. 6. Most eyes had a mean refractive cylinder of 0.50 D or less (Fig. 5), with no significant difference between the two postoperative assessments. This outlines the high refractive accuracy of both IOL designs and stability over this postoperative period, which is reflected in the high and stable unaided visual acuity outcomes already presented. A further analysis of how the refractive error alters over this time period was attempted in this study, with Table 3 outlining how the refractive error changed with 75.7% of EDOF eyes and 58.9% of trifocal eyes showing no change (< 0.25 D) in postoperative refractive error. Only 1 EDOF eye showed a change in refractive error of > 1.00D. This further outlines the refractive stability found with these IOLs; however, the difference in changes in refractive error over this time period needs further investigation.

This study also sought to assess QoV, functional vision and overall satisfaction through postoperative questionnaires at both postoperative assessments. Table 4 outlines the responses to the purpose-developed satisfaction questionnaire, where it was reported that 94.9% and 98.3% of patients would choose the procedure again or would recommend the procedure at the two respective postoperative assessments. Two patients reported a severe problem at distance at 1 month; however at 12 months they reported only a slight problem. Requirement for reading glasses (Table 4) is similar to that in a study of rotationally asymmetric multifocal IOLs [22]. Functional near and intermediate vision was excellent (Table 4) with no patients reporting a severe problem or worse at 1 year. Additionally, 93.1% of patients reported to be more than fulfilled or fulfilled when asked regarding how their expectations were fulfilled with the procedure at both the 1 month and 12 months postoperative assessments. This study appears to show a high satisfaction rating regarding functional vision. The mean overall daytime QoV score at 1 month was 9.12 ± 0.94 and 9.24 ± 0.78 at 12 months, which is superior to that found in other similar studies regarding bilateral implantation of multifocal IOLs [6, 17]. There was no significant difference between the two assessments periods. However, there was a significant improvement in the mean overall night score at 12 months (Table 5). A low incidence of bothersome side effects at both postoperative assessment was reported with most of the visual disturbances reducing at the second assessment; however none were significantly different. It appears that a high QoV and satisfaction is observed from the early postoperative assessment and is maintained within the first year. Patients who reported early postoperative issues improved 12 months postoperatively and were then satisfied with the outcome of the surgery. This study shows that this IOL combination provides high early postoperative satisfaction with a high QoV reported at 1 month, but it does appear that there is further improvement in nighttime QoV as patients may be neuroadapting over this period.

Two trifocal eyes required further laser enhancement. This was performed 11 months post-implantation with one patient to correct a residual refractive error of + 1.50 /—1.25 × 95 with the refractive error improving to + 0.25 D post LASIK. The second trifocal eye also received LASIK to treat a residual refractive error of + 1.00 / − 0.50 × 95 resulting in a post-LASIK refractive error of 0 D. Both patients noticed an improvement in their unaided vision. Eight eyes required Nd:YAG capsulotomy, which is significantly lower than a study of multifocal spherical IOLs [23].

In conclusion, this study found that the combined implantation of an EDOF IOL in the dominant eye and a trifocal IOL in the non-dominant eye provides excellent patient satisfaction and provides a range of clear vision up to 12 months postoperatively. A high overall night and day QoV score is reported at both time points, and it appears that neuroadaptation might have occurred between 1 and 12 months postoperatively, resulting in a significantly better overall nighttime QoV score. This study provides the clinician with information regarding combined EDOF and trifocal IOL implantation and how this combination performs up to 12 months postoperatively.

## Data Availability

Available upon reasonable request.
